# Integrating the complexity of healthcare improvement with implementation science: a longitudinal qualitative case study

**DOI:** 10.1186/s12913-022-07505-5

**Published:** 2022-02-19

**Authors:** Angela Melder, Tracy Robinson, Ian Mcloughlin, Rick Iedema, Helena Teede

**Affiliations:** 1 Monash Centre for Health Research and Implementation, Monash University, 43-51 Kanooka Gve, Clayton, Victoria 3168 Australia; 2 School of Nursing, Paramedicine and Healthcare Science, Charles Sturt University, Bathurst, Australia; 3 Monash Business School, Monash University, Melbourne, Australia; 4 Centre for Team Based Practice & Learning in Health Care, King’s College, London, UK

**Keywords:** Ethnography, Qualitative research, Health care organizations and systems, Quality improvement/report cards

## Abstract

**Background:**

Implementation science seeks to enable change, underpinned by theories and frameworks such as the Consolidated Framework for Implementation Research (CFIR). Yet academia and frontline healthcare improvement remain largely siloed, with limited integration of implementation science methods into frontline improvement where the drivers include pragmatic, rapid change. Using the CIFR lens, we aimed to explore how pragmatic and complex healthcare improvement and implementation science can be integrated.

**Methods:**

Our research involved the investigation of a case study that was undertaking the implementation of an improvement intervention at a large public health service. Our research involved qualitative data collection methods of semi-structured interviews and non-participant observations of the implementation team delivering the intervention. Thematic analysis identified key themes from the qualitative data. We examined our themes through the lens of CFIR to gain in-depth understanding of how the CFIR components operated in a ‘real-world’ context.

**Results:**

The key themes emerging from our research outlined that leadership, context and process are the key components that dominate and affect the implementation process. Leadership which cultivates connections with front line clinicians, fosters engagement and trust. Navigating context was facilitated by ‘bottom-up’ governance. Multi-disciplinary and cross-sector capability were key processes that supported pragmatic and agile responses in a changing complex environment. Process reflected the theoretically-informed, and iterative implementation approach. Mapping CFIR domains and constructs, with these themes demonstrated close alignment with the CFIR. The findings bring further depth to CFIR. Our research demonstrates that leadership which has a focus on patient need as a key motivator to engage clinicians, which applies and ensures iterative processes which leverage contextual factors can achieve successful, sustained implementation and healthcare improvement outcomes.

**Conclusions:**

Our longitudinal study highlights insights that strengthen alignment between implementation science and pragmatic frontline healthcare improvement. We identify opportunities to enhance the relevance of CFIR in the ‘real-world’ setting through the interconnected nature of our themes. Our study demonstrates actionable knowledge to enhance the integration of implementation science in healthcare improvement.

**Supplementary Information:**

The online version contains supplementary material available at 10.1186/s12913-022-07505-5.

## Background

Given the pace of technological advancement and growth in healthcare demand, governments are mandating healthcare transformation. Health systems are highly complex in their design, networks and interacting components and change is challenging to enact, sustain and scale. Recent evidence shows that healthcare improvement (HCI) is often delivered using simple methods that may lack rigour and efficacy [1–3]. Policy makers, academics, clinicians and those who deliver HCI at the coalface of healthcare, require greater insight into how transformative change can be enacted in complex systems, while at the same time, delivering HCI that is pragmatic and patient centric [1–3].

Implementation science (IS) brings rigour and evidence-based approaches to healthcare improvement, however it is a complex field involving many disciplines that bring different perspectives and often focus on generating theoretical concepts to advance academic understanding. This can contrast with the pragmatic need for “how to” approaches required to inform frontline healthcare improvement in practice [1–3]. Current IS frameworks can provide guidance for planning and undertaking improvement but more knowledge is needed about how to apply these frameworks to better understand how multi-disciplinary teams, embedded in complex improvement interventions, function over time, and how local adaptations and contexts can inform the spread and scale of HCI interventions [3].

Calls are increasing for integration between the IS and HCI to apply rigorous methods, and pragmatic approaches to improvement work [1]. The CFIR is used to design, implement and evaluate evidence-based interventions, and comprises five domains and 39 associated constructs [4]. The comprehensive nature of the CIFR makes it ideal for capturing the complexities of improvement work [5–8]. It encompasses: *intervention characteristics*: including perceived source and evidence strength and quality; *outer setting:* including community needs, resources and external policies or incentives; *inner setting*: such as perceived need for change and internal resources; *characteristics of individuals*: including knowledge and beliefs about the intervention, and *implementation process:* such as quality of planning and engaging staff.

Although widely used to plan and evaluate implementation studies, information on the use of the CFIR to evaluate complex, multi-faceted, person centred interventions is scant [9]. The CFIR can be seen as a determinants framework in that it can be applied with deductive reasoning to identify enablers and barriers to implementation outcomes. It is important to acknowledge how factors that influence implementation outcomes can manifest differently due to variations in health system structures, population cohort morbidities and resource availability [10]. This means that frameworks such as the CFIR may require adaptation and, while there has been significant growth in the use of the CFIR to support implementation research, missing elements, or limitations, of the framework have been identified, including sustainability and a focus on teams [10]. In addition, little is known about its application in pragmatic and sustained HCI. Some studies report difficulties translating the complex and sometimes repetitive construct definitions in the CFIR to fit their initiatives [8, 9]. Hence, using the CFIR lens, we aimed to explore how pragmatic and complex healthcare improvement and implementation science, can be integrated. We do so by examining the implementation process involved in delivering a complex healthcare improvement intervention*.* The implementation of the intervention, as a case study, involved integrating and evaluating routine mental health screening in a service providing antenatal care for refugee women. Details outlined in Additional file 2. This improvement intervention is driven by clear evidence that women of refugee background have an increased risk of mental illness during pregnancy that is compounded by pre and post settlement stressors [11]. Importantly, we aimed to gain greater insight into the process of the implementation of this intervention, not the specific details of the intervention, so as to provide insight from emergent themes for actionable knowledge to enhance effective and sustainable healthcare improvement and implementation.

## Methods

### Study design and data collection

This case study research was undertaken by the authors (AM, TR, RI, IM and HT) and was embedded within a larger ‘parent study’ investigating healthcare improvement at a system level with four public health services and a government department in Australia [12]. Here, we report findings from an in-depth longitudinal case study of an improvement intervention being implemented at one health service (Service P). The case study implemented an improvement intervention at Service P, the largest health service in its jurisdiction including six hospitals and highly diverse out-patient and community services, offering generalizability to a broad spectrum of larger health services, Additional file 2The improvement intervention was delivered by a Service P Implementation Team. To understand how to undertake pragmatic implementation and improvement in complex healthcare settings, we utilised exploratory and qualitative methods with ‘open-ended inquiry’ [13] using ethnographic observations of implementation team meetings, document reviews, and interviews with thematic analysis. The use of multiple methods allowed for an approach sensitive to context, participants, processes and behaviours and to explore the constructs and factors that have most influence on effective implementation and improvement [14–16].

The researcher (AM) was in situ throughout, allowing ‘real-time’ data collection in a ‘real-world’ context to limit retrospective bias [17]. Field-level participants included the implementation team, Additional file 2. Frontline clinicians (hereafter, referred to as target clinicians) who were expected to implement routine mental health screening, were excluded as our focus was on the “how to” of improvement work. We focused on the intervention implementation team actions, how they utilised resources, their interaction with diverse stakeholders and how they progressed the process of improvement.

**Fig. 1:**
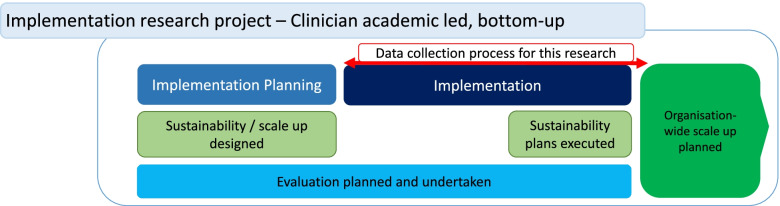
Data collection for this research (Includes Case Study implementation process)

Qualitative data was collected over 24 months (January 2017–December 2018). Semi-structured interviews (30–60 min) were completed with case study implementation team members involved in planning, implementing and evaluating the intervention. Document review was undertaken across internal project documents, meeting agendas and minutes, presentations and published literature (stemming from the case study) plus researcher field notes and unstructured observations of implementation team meetings. The data collection process is illustrated in Fig. 1.

Interview questions were informed by theoretical approaches of IS (particularly CFIR), complexity and improvement science [3]. General concepts explored in the interviews included constructs of leadership, context, process and content with the interview guide presented in Additional file 1. Interviews were audio recorded and transcribed. The data from interviews was transcribed, and along with field observations, was analysed using QSR NVivo 12 [18].

Themes were analysed progressively, until saturation was reached. Analysis was grounded and inductive, influenced by aspects of implementation, complexity and improvement science. Broad themes were elicited through an open-coding process [19], allowing first order constructs to be identified. This thematic analysis was undertaken by AM, TR and HT to minimise bias and substantiate themes and constructs that emerged. Themes emerged from the data as first order constructs, which were progressively collapsed into higher order second- and third-level constructs. A thematic structure emerged made up of main themes and related sub-themes reflecting the critical features of improvement work as it progressed, to achieve its aim of successful implementation. The conduct and reporting of this research was guided by the Consolidated Criteria for Reporting Qualitative Research [20].

The second phase of the analysis explored critical success features (themes) emerging from pragmatic ‘real-world’ improvement through the lens of the CFIR [4]. An analytic matrix was developed, juxtaposing our themes with CFIR components (domains and constructs) for a more in-depth understanding of how the CFIR components operated in a ‘real-world’ context. We also examined whether CFIR captured the observed pragmatic elements of this work. The mapping process identified commonalities, discord and revealed nuances across the CFIR. We aimed, here, to identify specific constructs that might enable better integration of HCI with implementation science to provide actionable knowledge to enhance effective and sustainable improvements in healthcare.

#### Characteristics of the case study

The case study is presented according to the CFIR constructs described in Additional file 2, also showing data collection timelines aligned with project progress. This provides an in-depth case description including the roles of implementation team involved, the process of improvement and the contextual issues that affected the work [4].

#### Case study outcomes

The intended improvement outcomes of the case study are presented in Table 1. This provides evidence that the case study intervention outcomes were achieved. That is, the case study intervention (the mental health screening tool and new model of care with referral pathways) was used in practice and feasibility and acceptability (of the case study intervention) was demonstrated. These outcomes are the results of the case study intervention, not the results of our research which was to examine the process of how the case study implemented their intervention.

**Table 1:** Improvement outcomes of the case study intervention

Case Study Improvement Outcomes	
• The implementation of a screening tool and associated processes was found to be acceptable and feasible for health professionals^a^ • From the perspective of patients involved, screening for mental health in pregnancy using a digital platform was found to be acceptable and feasible^a^	

^a^published work, authorship withheld to protect the identity of participants and the heath service

## Results

Our ethnographic case study collected data from 18 interviews, 16 non-participant meeting observations and 16 examined documents.

### Thematic analysis

Main themes emerged on “how to” undertake pragmatic implementation in a complex healthcare setting across:

- Leadership: characteristics of the team leading the improvement work
- Application of an evidence-based research process, with pragmatic iterative action to ensure improvement work progress (this included designing and planning for sustainability and scale) and
- Navigating context (local and broader issues, organisational and local settings, aspects of the clinical condition) that affected the improvement work

Table 2 outlines the main themes and sub-themes and the inter-related dynamism across these. The team displayed leadership qualities of agency and collaboration engaging clinicians as they navigated a shifting and complex context, while applying scientific thinking with pragmatic, responsive and iterative action. Table 3 provides example quotes for each theme, and indicated as Quote (Q) 1 to 11.

**Table 2:** Thematic Analysis and main themes

Main Themes	Leadership	Processes applied	Navigating Context
**Sub-themes**	• Agency • Capability for engagement • Teamwork and collaborative approaches	• Focus on patient need • Planning, execution, evaluation - Designed for sustainability and scale-up - Theory driven improvement and implementation - Iterative • Project management	‘Bottom-up’ approach - Embedded at the point of change - Co-designed

**Table 3:** Exemplar responses illustrating the major themes: Leadership; Process; Context

**Leadership and engagement** (Quote (Q) 1) I think having champions is really useful, so having people who are - and they have to be the right people, because it’s not necessarily going to be the senior leader, it’s someone who is respected within the space, who people listen to, who isn’t necessarily the named leader - and engaging them in a meaningful way and then getting them to lead the change. So I don’t think we necessarily need the senior leaders or the whole units at the table, but we need selected important people to be engaged and be able to be seen to be engaged so that they can take it forward. But I think we do need everyone, and I think there are so many units that if we don’t give the opportunity, at least, for each of them to be engaged then they can be lost. If it’s just engaged at a program level there are an awful lot of people who sit under the same banner and have really, really diverse practices and workforce and everything else, so being able to have representation from areas is, I think, going to be important at the outset, rather than just bringing them in once it’s been decided. **Senior Medical Lead** (Q2) I think it’s a very collaborative process, because...Mostly people aren’t, people are pretty happy to work you know and I think there may be times in the future where for example, [X] and [Y] are interested in following up some of the children and I would be happy to hand that over, that’s their areas of expertise and I don’t think, I think we all recognise each other’s area and we are all building to each other’s strengths. And so far there hasn’t really been any competiveness. **Clinical Lead and senior academic** (Q3) We spoke to settlement services, community members, the managers and staff I worked with at the community health centre, because you want to look at where you’re going to get your referrals from. People need to know about the service, they need to know what’s happening and how it’s going to be implemented. Feel that they’re actually a part of the process, not just left out - through meetings, chatting. I think essentially I feel I’m a good networker, and I think that’s something that - when I think back to that work we did in the refugee work and this as well, I’ve also been really fortunate, I’ve worked in [X health service] for 30 years so I know a lot of people, the midwifery staff know me, I know them. I’ve worked with a lot of them. So all those things have helped a lot too. And because, in the sense I’m one of them, that’s probably helped. **Project Officer**	
**Process of improvement and implementation** (Q4) Before we even did the formative research, the important thing was we knew we needed to speak to community members **Senior Research Fellow (Psychology)** (Q5) The main driver was that it was a very high risk population and we were concerned about that gap and care for them. So as we went through we started talking to more and more people, we met more people. And then we met the CEO from a not for profit non-government organisation that has funding to provide to try and introduce screening in pregnancy for anxiety and depression and they already had – so they had tools and resources and experience that we could leverage off. **Senior Research Fellow (Health service research)** (Q6) I contacted the maternity services, found out why they weren’t doing it. Looked at what could we do that would enable us to try it and then the important thing for us was you know, before we even did the formative research, the important thing was we knew we needed to speak to community members. Because a lot of people anecdotally have always said that you can’t scene with cultural and linguistically diverse women or women from those backgrounds. Because the screening tool doesn’t work with them, because they have different concepts of mental health and therefore they won’t want to engage with it. But that wasn’t the message and that’s why we really made an effort too. **Implementation team member** (Q7) The national guidelines are for every woman. We decided to start with refugee women, acknowledging that it was a high risk population and yep, probably where the greatest unmet need was. Of course it was also the most complex population which is one of the reasons why it hasn’t been done. And there was a little bit of an attitude of well if we can make it work in this population we can probably make it work in the general population. So the chances of being able to roll it out across all of the Maternity Service would be great if we could get it to work in this most challenging circumstance. If we can demonstrate that it works in this situation then there can be very little criticism or very little but what if? There are very few excuses that can come up that we have not already seen and addressed. **Senior Research Fellow (Health service research)** (Q8) We need to prove the effectiveness of the assessment tool, before we set about sustaining it in practice. If it wasn’t effective at achieving the set objective then we would not want to sustain it. **Senior Research Fellow**	
**Context** (Q9) We’ve received funding from [X health service] and [X university] and from [X research translation centre] as well. And the leverage that then gives us is that if we experience really serious barriers we can go to very high levels at those organisations who hold quite a lot of power and say, “Look, you and these other organisations have invested considerably in this project, recognising that it is a key priority for you, and we are experiencing these problems that we haven’t been able to address ourselves and we need some high level support on it.” And we’ve not had to use that because it’s quite a blunt instrument. Yeah we haven’t had to really call that into use yet but it’s nice to have that strategic high level support. **Senior Academic Lead** (Q10) I can’t speak highly enough of the people above me. I think that they really are cognisant of the impact of perinatal mental health on women and newborn well-being, and they’ve been very keen to explore opportunities to do things differently or to do some short sharp, change management as an intervention that might make a difference to the outcomes that we’re getting. Yeah, so certainly at the levels that I’ve been, they’ve been very engaged and very curious about what we can do and have been more than supportive. **Midwife - Nurse manager** (Q11) But it’s got to the point where a lot of the hard work has been done. But I think some of that has been because I’ve been quite strategic. I’ve been around long enough to know that research is something that buys you credibility in academic environments. And to be strategically aligned with projects like this, or other projects, buys me credibility, in terms of, you know having bargaining power and having some influence, I suppose. **Service manager**	

### Main theme – leadership: characteristics of the implementation team leading and engaging with target clinicians with improvement work

This main theme and sub-themes captured the implementation team’s demonstration of diplomacy and communication required for interaction and engagement with those involved in healthcare improvement. The sub-themes included agency, capability and teamwork. (Q1 to Q3).

#### Agency

Agency is the capacity to act with purpose, power and courage to initiate improvement in response to gaps or suboptimal quality of care [21]. Agency was exhibited in response to patient need, reinforced by national guidelines. The implementation team recognised the relevance and importance of aligning internal organisational strategic directions and external levers, such as national guidelines. The team utilised this structural lever to initiate dialogue with stakeholders, who brought expertise to the improvement work from within the health service and externally. It galvanised the belief in the work and the desire to improve with clear the rationale for immediate action from stakeholders, such as fellow clinicians, health service personnel and organisational leaders, as champions. The team identified and engaged with others with additional expertise and a shared vision. The implementation team’s actions revealed passion, competence and expertise, with confidence to act and lead. As leaders of change with agency to drive the work, the team recognised and leveraged their expertise, position and role. This was a significant characteristic, consistently displayed throughout the work and with all stakeholders.

#### Capability for engagement

Diplomacy, or high-level communication skills were applied to achieve engagement and negotiation with target clinicians. The team worked hard to connect, understand and engage with clinicians around the new practice considering context and barriers and enablers of the intervention and its implementation. They adopted collaborative, shared leadership, to adapt, modify and shape the process, according to contextual issues, such as time limits, patient needs, or information technology capacities. The team consistently inspired others using strong communication skills, achieved through regular meetings between the implementation members and target clinicians within the setting where the intervention was delivered.Emotional intelligence and diplomacy skills, tacitly demonstrated and explicitly described by the team were recorded. Tacit characteristics included engagement with target clinicians within a clinical setting to gauge their reactions and to respond to unspoken messaging And also evidenced in observations of team meeting discussions. This quality reflected personal motivations of the implementers and the leveraging of a shared motivation with target clinicians to achieve “best practice” for patient care.

#### Teamwork and collaboration

Teamwork was a dominant characteristic and was connected to aspects of networking, negotiating, relationship-building and connection development. In terms of explicit characteristics, strong teamwork principles of collaboration and co-design were applied including frontline managers and clinical teams (target clinicians). Implementers worked hard to build connections and relationships between target clinicians and improvement work, including intervention co-development and refinement. Communication and connection-building served to foster trust and enhance relevance of the improvement work with target clinicians. The team communicated consistently and frequently with all target clinicians, as observed in team meetings and through interview discussions with case study implementation team members.

Collaborative approaches included problem-solving, where no problem was too insignificant or to intractable. Frontline teamwork was demonstrated through consistent target clinician engagement and on-ground coaching, demonstrating high levels of communication with frontline staff and recognition of on-ground problems and progress.

### Main theme - process of improvement and implementation

This theme highlights key motivations for the improvement work and the structural and practical elements of implementation team action. It includes team processes utilised, and actions taken to progress the work. Sub-themes included the focus on patient need as a key motivator for clinicians and the planning, execution and evaluation of the implementation process. Other sub-themes involved intervention development, theory-driven implementation processes, consideration of sustainability and scale up issues, analysis of implementation barriers, enablers, and measurement and use of process and outcome evaluation. (Q4-Q8).

#### Method of improvement and change process (planning, execution and evaluation)

Observation of the method of improvement and change process applied by the implementation team was a key aspect of our research. The Case study methods involved a structured approach containing key activities included planning, execution and evaluation, are outlined as “Process” in Additional file 2, Fig. 1 illustrates the these process. Driven by a national guideline [22] the case study implementation team sort to implement ‘best practice’ using a complex intervention (Additional file 2) The case study strongly focused on sustainability and scalability, once proven effectiveness was proven. This team applied an established implementation theoretical framework (the Normalization Process Theory) [23] that underpinned evaluation and measurement of practice change and health outcomes.

In studying the process of implementation, we observed case study implementation team undertake in-depth assessment of patient needs and clinician perspectives to inform the co-designed improvement process. The team reported (and published) extensive communication with multiple stakeholders internally and externally to the health service, before and during the implementation. Iterative co-design with target clinicians, clinical leaders, technology experts and academics occurred throughout. Modifications and solution development occurred more intensely at the beginning and less so over the implementation. Coaching with target clinicians was also intense at the beginning to ensure feasibility and practical use of the newly implemented assessment tool. While the screening tool was designed for sustainability and spread, it was an additional task to usual practice. To this end, sustainability was considered and planned from the outset, but strategies were only instigated after evaluation indicated efficacy. Several implementation team members indicated, “If we can get it right in this setting [refugee, maternal health services], it should be easier to establish it in a less challenging general maternity setting”.

A prevailing observation was the unremitting effort and the availability of the project officer and clinical leaders (both part of the team) for clinicians adopting new practices and tools. All team members, particularly the project officer, were readily available to observe, coach and engage frontline target clinicians as well as acting as liaison between these clinicians and the implementation team.

The recognition of patient need was demonstrated through the clear commitment to ensuring this worked for the target clinicians and of prime importance, for the refugee women. Considerable effort was committed to developing and refining the screening tool to ensure it was understood by the women, across terminology, cultural appropriateness and translation into different languages.

#### Project management

Project management was an important role for the improvement/change facilitator, who was also a coach and PhD Scholar, with a background as a midwife and maternal child health clinician. Tasks involved organising meetings, progressing the project and reporting updates on all aspects of project progress. This was a regular and ongoing task to articulate and investigate problems and trouble-shoot and resolve situations that reconciled both research purposes with pragmatic actions.

### Main theme - navigating context

This theme captured diverse aspects of the case study context and how the implementation team navigated this. Sub-themes included project governance at a local and organisational level, and the team positioning, allowing multi-disciplinary capability, to respond to a changing complex environment, adjusting iteratively. (Q9-Q11).

#### Governance

Although governance represented a ‘bottom-up’ approach informed by implementation research, co-design and a collaborative approach to improvement, leadership and support ‘on the ground’ came from local leaders directly involved in service delivery with the identified vulnerable population and improvement setting. The senior clinical lead in the implementation team engaged with progress and problems with the Department head and manager, to secure ongoing support including for sustainable implementation.

Team members had roles that straddled an integrated Research Translation Centre (RTC) or “implementation laboratory”, firmly established as a partnership across the university and health service [24]. The team were also largely clinicians and central stakeholders in the health service and improvement process. This leveraged the onsite partnership between the RTC and the health service. Team members often wore two hats, as implementers and clinician leaders. This research lens and expertise facilitated insightful perspectives about the improvement process, balanced with practical implementation ‘at the coalface’. Additional academic funding was attracted to support the project, while senior researchers undertook the work as part of their academic roles. The clinician leaders in the implementation team also undertook the work as part of their role in delivering high quality care.

### Mapping our themes with CFIR

To enhance understanding of the “how to” issues of pragmatic implementation and improvement in complex healthcare settings, we mapped themes that emerged from our longitudinal ethnographic research of an improvement case study to the CFIR constructs to better understand how a real-world improvement could integrate with IS. We aimed to bring further depth to understanding the process of IS and HCI. The mapping process revealed strong alignment, limitations and enhancements to CFIR in the following ways.

Leadership and process themes from our case study cut across the CFIR constructs and domains, this is unsurprising given our research focus was on the process the implementation teams used, and these CFIR components reflect much of the contextual and process aspects that impact implementation work. Patient need while included as domain in the CFIR, our case study illustrated the significant influence it played in our case study. An identified limitation between our process theme and CFIR was that of sustainability and scale-up. While evaluation and reflection of progress (in CFIR) was an ongoing activity, our case provided further granulation about the process of design and execution to ensuring sustainability. The complexity of contextual issues aligned strongly with several CFIR context constructs and domains, focussed mainly on the outer and inner settings.

The CFIR identifies five constructs essential to implementation success (inner setting, outer setting, etc.). Our themes aligned with all five constructs, presented in Table 4, however, our themes of *leadership* (particularly distributed models of leadership was persistent, iterative and attentive to collaborative engagement with stakeholders) and *process* cut across all CFIR constructs. The implementation team navigated and embraced the complex and dynamic contextual circumstances, as well as the intervention development and implementation process itself. Our research reflected an inter-related nature of the themes and constructs, reflecting the dynamic aspects of the improvement work. Our leadership theme, in particular, aligned to all aspects of the CFIR, through agency, engagement and skills. Furthermore, it aligned to other CFIR constructs; Outer setting (B. Cosmopolitanism), Inner setting (Networks and Communications) and Process (B. Engaging) (Table 4). In this distributed leadership model of our case study, no one person held all responsibility and the team collaborated, reflecting individual capabilities and responsibilities, and led the work by engaging others and navigating context. This provides insights into a leadership model that appears to enhance implementation success. Rather than articulating anything missing in the CFIR, the mapping activity provided depth and demonstration of the inter-connection of CFIR constructs.

**Table 4:** Mapping of themes with CFIR constructs and domains

Our themes	CFIR Constructs & Domains				
	Outer setting	Inner setting	Characteristics of individuals^**a**^	Intervention characteristics	Process
**Leadership**	B. Cosmopolitanism	B. Networks and Communications E. Readiness for Implementation- E1. Leadership Engagement			B. Engaging B1. Opinion Leaders B2. Formally Appointed Internal Implementation leaders B3. Champions B4. External Change Agents
**Context**	A. Patient needs & resources B. Cosmopolitanism D. External Policies & Incentives	B. Networks and Communications D. Implementation Climate • D3. Relative priority E. Readiness for Implementation • E1. Leadership Engagement • E2. Available Resources	A. Knowledge and Beliefs B. Self-efficacy		
**Process**	A. Patient needs & resources	D. Implementation Climate • D4. Organisational Incentive and rewards: • D5. Goals and Feedback • D6. Learning Climate. E. Readiness for implementation: • E3. Access to information and knowledge		C. Relative advantage D. Adaptability F. Complexity	A. Planning C. Executing D. Reflecting and Evaluating

^a^Characteristics of individuals not directly observed by the researchers, but discussed by the implementation team and observed in their actions toward/with the target clinicians

In terms of applying CFIR to pragmatic and sustained HCI, the reality is that all of the elements of complexity or implementation science are at play in a real-world implementation setting and CFIR enables the synthesis of many complex factors that focus on, and impact, the processes of implementation.

The mapping provided an in-depth examination of the CFIR domains and constructs in light of a real-world circumstances. This revealed the complex interplay of factors operating in healthcare improvement work, highlighting the critical nature of relationships between the constructs, and presenting a complex nuanced assessment of how implementation teams interact with stakeholders and contexts and iterative processes that underpin and confront change at the clinical frontline.

## Discussion

There is a clear need to optimise approaches to deliver effective and sustainable improvement in health care, integrating methodological rigor and theory driven implementation science with pragmatic healthcare improvement methods [1, 6, 8, 9]. In this context, our research reports three main themes from the improvement work; leadership, context and process. Our study highlights the fluidity of CFIR constructs and how they overlap and are interconnected. At the same time, this study provides a more nuanced understanding of the CFIR constructs and the integral role of leadership and team work that cut across all domains.

### Leadership and engagement

While leadership engagement is included in CFIR we bring a more nuanced and pragmatic understanding of leadership to the framework. Our research provides an in-depth demonstration of what leadership of improvement looks like and how these leaders enacted the process of improvement with stakeholders. Key leadership capabilities included agency to lead the work and capability to engage and communicate about the improvement process with target clinicians and to facilitate the process continuously with them, learning iteratively together.

The importance of leadership was highlighted by Damschroder 2009 [4] and the need to build a cohesive team consisting of effective champions and stakeholders, who are most likely to make the implementation a success. Here, the multidisciplinary implementation team demonstrated leadership through the agency, skills and capability to engage with target clinicians.

A recent integrative evidence review [25] described attributes of effective facilitators involved in healthcare improvement, with key qualities aligning to the leadership displayed by the implementation team. Ellegdge 2019 reported that self-awareness, self-management, social awareness, relationship management, skills, and knowledge translation and understanding represented key competencies for facilitators to effectively influence success of knowledge translation to improve practice [25].

Illot 2012 [5] noted that the issue of leadership in the CFIR is under-developed and could potentially go further to describe the connection of leadership with other constructs of implementation and describe how this component continually interacts with the stakeholders involved in the improvement process and context in a pervasive and active manner. Leadership is a complex and critical factor of implementation and improvement work, is relevant to context and process and requires more in-depth framing and investigation that captures the breadth of its influence in future research and healthcare improvement efforts.

### Context

Our context theme encompassed both aspects of the CFIR inner and outer setting. These CFIR constructs reflect some granulation that was illustrated in our case study research. However, CFIR could go further to emphasise the aspect of ‘patient need’ influencing the implementation process, and the significance of it as a critical motivator for target clinicians to participate in taking up new practices to benefit patients.

In terms of outer setting constructs, patient needs, especially in vulnerable groups, provided a critical trigger for the improvement work and galvanised all involved. In addition, an external best-practice guideline provided an incentive to implement evidence-informed practice.

Our context theme incorporates patient needs which is contained within an outer setting domain of CFIR. Our research emphasised the significance of this important driving factor in the consistent inclusion and reflection throughout the improvement process. It was a key trigger for the improvement work, and was of paramount importance to the implementation team to meet women’s mental health needs. It was highly relevant for the women involved in the process and the feedback they provided with respect to their support needs, and it dominated the purpose for delivering high quality care from the perspective of the target clinicians. Focus on patient need was woven throughout, actively and passively with the women and target clinicians. Illot 2012 [5] and Safaeinili 2019 [9] undertook a validation process which examined the comprehensiveness of CFIR with healthcare improvement projects and highlighted this as an underrepresented aspect and a gap in the framework. Safaeinili 2019 [9] goes so far as to suggest that patient involvement should be an additional domain to the CFIR. Our work would suggest that patient need and engagement enhanced clinician engagement and was vital in this improvement work.

Furthermore, our context theme captures the governance of bottom-up approaches to improvement work. Cosmopolitanism and networks were reflected in this bottom-up approach, and the partnership between the implementation team members, made up of research and clinician experts embedded within a RTC that was externally positioned to maintain knowledge exchange, and focus to progress the work, combined with internal members (local leaders and champions) who were established and connected within the local healthcare setting as clinician managers and service directors [26]. This contextual fit supported and enabled the team to progress the improvement work, while still providing required care and aligning other local priorities. Having a team that was present at the point of change is also emphasised in research by Bonawitz 2020, who highlights this embedded aspect of having change champions who understand the practicalities at the frontline and who can leverage organisational influences, or resources, to facilitate the process to achieve the envisaged change [27].

There is increasing recognition of the complexity of healthcare delivery and the need for improvement work to acknowledge that to achieve success in a complex setting where a responsive, bottom-up approach is better suited [2, 28–30]. Our case study demonstrated local involvement in refining the approach and when this was fostered (through the implementation team), the impact was positive (in terms of practice change). The attentive response of the implementation team to local issues, enabled a stronger feedback mechanism with “grass-root” creativity to resolve improvement process problems. An agile, attentive and engaged leadership demonstrated by the implementation team, leveraged networks within and external to the setting, communicated with each other and with clinicians about the improvement work, and connected extensively with patients around the new practices. McCullough 2015 reinforces this finding and identified key contextual elements, such as leadership, teamwork, and communication, interacting with each other and contributing to site-level uptake of the evidence-based practices [31]. Here we observe the presence of these important factors affecting the uptake of introduced practices. Further research is needed to provide insight into how specific characteristics of context, particularly the application of a bottom-up approach, can influence improvement outcomes.

### Process

Damschroder 2009 [4] reports that process is the “single most difficult domain to define, measure, or evaluate in implementation research”. Our theme of process amalgamated CFIR intervention characteristics and process. We did this because intervention characteristics, patient need and clinician requirements influenced our process theme. The iterative, interactive and collaborative process used by the implementation team, aligned with the components in these CFIR domains and constructs. These processes conferred through the leadership of the implementation team and engaged stakeholders in the vision, ensured adaptability and addressed complexity to safeguard success across the planning, execution and evaluation phases. Furthermore, the process consistently considered and addressed patient and clinician needs and resources, provided and discussed shared goals, and updated progress in an iteratively collaborative learning scenario to identify and improve where needed. The team were not deterred by uncertainty; rather they demonstrated confidence in each other’s expertise, as well as the expertise and feedback of the target clinicians they were working with to implement a new practice and tool, and in doing so, further enhanced and fostered engagement of all stakeholders in the improvement work.

An identified limitation between our process theme and CFIR was that of sustainability and scale-up. While evaluation and reflection of progress was an ongoing activity, our case set out to design an intervention and implementation strategy for sustainability and scale-up. The case study process involved activities to ensure sustainability and scale-up, which is not incorporated explicitly in the CFIR. Ilott 2012 also identifies this gap and points out the limitations of the ‘reflect and evaluation’ domain, highlighting the need for further definition and investigation on how to capitalise on organisational change efforts [5].

### Limitations and strengths

The limitation of including a single health service in a public universal healthcare setting may limit generalisability. However this health service was part of a national public health system and this was one of the largest services with broad reach. The case study itself was designed for and is now being scaled broadly and has been independently evaluated by the study team and shown to be acceptable and scalable. With scale-up, evaluation will provide insight into broader implementation.

Here we retrospectively applied the CFIR, acknowledging that this is designed for prospective use to guide implementation research. However, it is based on learnings of what works in implementation and by its nature was in part constructed from retrospective evaluations such as this. Here our aim was to provide insight into the CFIR components, and how they may interact with each other. Prospective application would not have aligned with the ethnographic approach of observing real-life work, rather than primary case study implementation research unfolding.

We acknowledge that our approach of the in situ researcher has limitations including potential bias that might have influenced the process of the improvement work. However, it is precisely the strength of the longitudinal immersion and insider status that enabled this research and provided the insights into the normally overlooked deeper aspects of the context in which HCI takes place. As the case study scales further evaluation will enable further evaluation above and beyond that from in situ researcher, although it will lack the depth of such an approach.

## Conclusions

This research on a ‘real-world’ healthcare improvement case study using the CFIR lens, provides an in-depth and rich understanding of integrated IS and pragmatic HCI. It provides an illustration of the cross-interaction of the components, and presents a nuanced picture of CFIR and the implementation processes that it aims to guide. Key themes included leadership, context and process, which mapped closely to the CFIR. Specific findings include the vital role of leadership agency to cultivate relationships with target frontline clinicians, engaging them in the process of improvement, enhancing participation through planning, execution, evaluation and sustainability at the frontline. The importance of leaders with clinical and rigorous implementation expertise emerged, as did engagement of multi-disciplinary cross-sector support to manage complexity, contextual issues and delivery on the shared vision of improved outcomes. The vital role of stakeholder engagement and co-design emerged, with patient and clinician need key throughout the improvement work. We highlight the opportunity to integrate sustainability and scalability, not currently explicit in the CFIR, yet fundamental to pragmatic healthcare improvement. Overall, applying the CFIR lens, we produce actionable knowledge to enhance integration of implementation science and pragmatic health care improvement, to improve practice and patient outcomes.

## Supplementary Information

**Additional file 1.** Semi structured interview guide questions.

**Additional file 2.** Characteristics of the case study using the CFIR constructs.

## Data Availability

The datasets used and/or analysed during the current study are available from the corresponding author on reasonable request.

## Abbreviations

CFIR: Consolidated Framework for Implementation Research
HCI: Healthcare improvement
IS: Implementation science
AM: Angela Melder
TR: Tracy Robinson
IM: Ian Mcloughlin
RI: Rick Iedema
HT: Helena Teede
RTC: Research Translation Centre
