# Supplementary material for: Integrating the complexity of healthcare improvement with implementation science: a longitudinal qualitative case study
Source: BMC Health Serv Res. 2022 Feb 19;22:234. doi: 10.1186/s12913-022-07505-5 (PMC8858551; doi:10.1186/s12913-022-07505-5)
Supplement: Supplementary file 1 — Additional file 1. Semi structured interview guide questions. [file 12913_2022_7505_MOESM1_ESM.docx]

**ADDITIONAL FILE 1. SEMI STRUCTURED INTERVIEW GUIDE QUESTIONS**

**Research Title**

Exploring organisational innovation, redesign and implementation for sustainable healthcare improvement at scale

**Research Questions**

What factors, practices and relationships exist that provide an understanding about sustained organisation-wide innovation, redesign and implementation within a health service?

**Interview Questions**

***Question list***

This interview will cover 4 aspects of work you are involved in:

1. Planning the innovation or change
2. Implementing the innovation or change
3. Measuring the change
4. Sustaining and scaling the change
5. General learnings on the change processes

The questions listed below are guide questions to be used within a semi structured interview process.

| **PLANNING THE CHANGE**  **How did you come to the decision to implement what you did?**  *Alternative q: Could you please describe what it is you are implementing?*  **Can you describe the process of planning?**   - How did you engage with the research partners? Tell me about the partnership/steering group that leads the work? - How did you engage with the health service? - Did you use a structured approach? - Aspects to probe for: vision, Leadership (support), resources, capabilities, structural issues, capacity of organisations involved, capability of implementers to undertake the work, measuring/monitoring change, scaling and sustainability issues.   **What are the governance and leadership structures for this multi partner, multidisciplinary, co-designed project within the partner and [X] health service?**   - Why are these individuals/organisations involved at this level? - Who is the key champion(s) for this project? - Why are they the champions? How did it happen?   **What financial issues been considered?** |
| --- |
| **IMPLEMENTING THE CHANGE**  **Has there been much difference in terms of what you planned, compared to the actual experience of implementation?**   - What are some of the context and cultural issues you have encountered? - How did you navigate them? Did they impede/support you? - What barriers have you experienced? (Were these foreseen or unexpected?) - Have you had to modify anything to the intervention/bundle of care/care pathway/guideline for [X] health service implementation? - How? What? Why?   **How has the engagement in planning phase impacted the roll out or implementation phase? (wrt leaders/implementers/target community)**   - How have leaders in the organisation and partnership responded to the roll out/scale up of the initiative? - What impact did their response have on the project?   **Can you please tell me about the communication plans about the project and intervention that is being implemented?**   - Aspects to probe for: vision statement, Leadership, resources, capabilities, structural issues, capacity of organisations involved, capability of implementers to undertake the work, measuring/monitoring change, scaling and sustainability issues.   **Tell me about the communication strategy you planned, did it go as planned?** |
| **MEASURING THE CHANGE**  **How is monitoring and evaluation of the roll out of the initiative or the impact of the initiative being done?**   - Data management: How is this addressed? - Is there a need for patient data linkage? (Within [X] Health service or further – check for scale up planning of data management) |
| **SUSTAINING AND SCALING THE CHANGE**  **How will you sustain the initiative at [X] health service?**  **Will you scale to other sites at [X] health service or other service sites?**   - Has sustainability and scaling up processes considered organisational structures, co-ordination of action and performance monitoring and evaluation? |
