# Supplementary material for: Integrating the complexity of healthcare improvement with implementation science: a longitudinal qualitative case study
Source: BMC Health Serv Res. 2022 Feb 19;22:234. doi: 10.1186/s12913-022-07505-5 (PMC8858551; doi:10.1186/s12913-022-07505-5)
Supplement: Supplementary file 2 — Additional file 2. Characteristics of the case study using the CFIR constructs. [file 12913_2022_7505_MOESM2_ESM.docx]

**Additional file 2.** Characteristics of the case study using the CFIR constructs

| **CFIR constructs** | **Case Study Characteristics** |
| --- | --- |
|  | **Case Study Improvement Aim:** To integrate routine mental health screening (as recommended in the Mental Health Care in the Perinatal Period: Australian Clinical Practice Guidelines) (22) into antenatal care for refugee women and link outcomes of the screening tool to a referral pathway to community care |
| **Outer Setting** | National guideline recommending ‘best practice’ / External funding / Commercially developed tool to be implemented |
| **Inner Setting** | - The improvement project was undertaken within a specialised clinic, situated within a large publically funded teaching health service. This health service consists of five hospitals and several diverse community-based services. It is the largest health service at a state level jurisdiction - Executive suite and departmental heads and program directors all changed throughout the time of the case study. This also included the resignation of the Director of Organisation Innovation - The Implementation Team included multidisciplinary members (clinical and non-clinical) |
| **Process (Includes Intervention Characteristics)** | - The project was initiated through identification of an evidence-practice gap and pervasive patient need - The project was delivered in a perinatal clinic for refugee women   Bottom-up, co-designed improvement research:   - The project was led by a partnership Research Translational Centre, external to direct care delivery, yet integrated with the health service and clinical personnel through formal legal partnership, joint funding and staff who occupy dual clinician research roles - Academics, clinicians, patients, external experts, clinical managers and service directors administered the project and constituted the Implementation team. - Extensive stakeholder input included patients, academics, technical experts, and clinicians - External funding was secured for project work, intervention development and implementation team support - Hospital departmental support was secured with regular updates and ongoing engagement and dialogue - Health service executive support was secured but remained at arm-length and did not engage in the improvement work. |
|  | - Delivery of the intervention occurred in a maternity service and involved co-ordination with external serviced for refugee women - Implementation followed the Normalization Process Theory (23), with implementation, refinement, evaluation, sustainability and scale-up considerations embedded from the beginning. Once efficacy and effectiveness were achieved, both sustainability and scale-up activities were planned and are now underway - The improvement work involved the implementation of a complex intervention that included:   - A multidimensional perinatal mental health-status assessment tool using an online platform   - Intensive education and coaching about using the online tool. The tool was translated into different languages for patient use, reflecting literacy needs of the vulnerable target population   - Linkage of the screening tool outcomes to a referral pathway to community care - This research project followed the case study until it reached scale-up stages |
|  | Refer to Figure 1 for an illustration of the implementation processes adopted by the case. |
| **Personnel involved undertaking the improvement work - The Implementation Team members** | Implementation Team members:   - Project Officers* - Senior Research Fellow (Health service research)* - Senior Research Fellow and Obstetrician (Service P)* - Senior Research Fellow (Psychology) - Midwife - Nurse managers - General Practitioner - Service Manager (Service P) - Senior Research Fellow and Midwife (Service P)   *Key roles:   - Project Officers: PhD Scholars with clinical background (midwife, maternal child health clinician, psychologist) - Senior Research Fellow (Health service research) - Senior Research Fellow and Obstetrician   The key roles in the Implementation Team were occupied by personnel who operated within an integrated Research Translation Centre and the implementation approach was consistent with identified best practice in translational research to achieve impact [24] For example, the intervention was triggered through identification by researchers of an evidence‐ practice gap and pervasive patient need. This identification drew on research indicating that perinatal depression and perinatal anxiety affects up to 20% of all women in pregnancy during the first twelve months post birth with debilitating effects on women, children and families (De‐ identified Published Paper). In seeking to address this gap, Centre researchers partnered with clinicians within Hospital P and implementation and innovation experts to address the issue. In addition, extensive input was actively sought from diverse stakeholders including patients and community, academics, technical experts and clinicians.  The composition of the Implementation team reflected this ethos with members with appropriate skills and expertise drawn from a clinical research background (Senior Research Fellows, PhD Scholars, front line clinicians (nurse managers and midwives, maternal child health clinician, psychologist, service manager and obstetrician from Service P) as well as a General Practitioner. This cross‐ disciplinary team was managed through a governance framework that included all key internal and external stakeholders |
| **Characteristics of individuals involved** | - Project delivered to a tightly defined clinical setting and highly complex and vulnerable patient group. - Clinical managers and front-line clinicians delivering care in the perinatal setting were involved in taking up the new practice, collectively referred to “the stakeholders” |
